# Phosphorylation of XIAP by CDK1–cyclin-B1 controls mitotic cell death

**DOI:** 10.1242/jcs.192310

**Published:** 2017-01-15

**Authors:** Ying Hou, Lindsey A. Allan, Paul R. Clarke

**Affiliations:** Division of Cancer Research, School of Medicine, University of Dundee, Jacqui Wood Cancer Centre, Ninewells Hospital and Medical School, Dundee, Scotland DD1 9SY, UK

**Keywords:** Mitosis, Apoptosis, Cell death, Caspase

## Abstract

Regulation of cell death is crucial for the response of cancer cells to drug treatments that cause arrest in mitosis, and is likely to be important for protection against chromosome instability in normal cells. Prolonged mitotic arrest can result in cell death by activation of caspases and the induction of apoptosis. Here, we show that X-linked inhibitor of apoptosis (XIAP) plays a key role in the control of mitotic cell death. Ablation of XIAP expression sensitises cells to prolonged mitotic arrest caused by a microtubule poison. XIAP is stable during mitotic arrest, but its function is controlled through phosphorylation by the mitotic kinase CDK1–cyclin-B1 at S40. Mutation of S40 to a phosphomimetic residue (S40D) inhibits binding to activated effector caspases and abolishes the anti-apoptotic function of XIAP, whereas a non-phosphorylatable mutant (S40A) blocks apoptosis. By performing live-cell imaging, we show that phosphorylation of XIAP reduces the threshold for the onset of cell death in mitosis. This work illustrates that mitotic cell death is a form of apoptosis linked to the progression of mitosis through control by CDK1–cyclin-B1.

## Abstract (precis)

**Summary:** Phosphorylation of XIAP by CDK1–cyclin-B1 controls the onset of mitotic cell death, which is important for surveillance against defects in mitosis and the response to cancer drugs that target cell division.

## INTRODUCTION

Anti-cancer drugs such as microtubule poisons arrest or delay cells in mitosis due to the action of the mitotic or spindle assembly checkpoint. This checkpoint normally prevents anaphase from occurring before all chromosomes are correctly bi-orientated on the metaphase spindle by inhibition of the anaphase-promoting complex or cyclosome (APC/C), a large E3 ubiquitin ligase complex that targets mitotic regulators such as securin and cyclin B1 for destruction by the proteasome (Primorac and Musacchio, 2013). Cells that are arrested for a prolonged period in mitosis can undergo cell death through the process of apoptosis (Allan and Clarke, 2007; Gascoigne and Taylor, 2008). The propensity for apoptosis is increased by the duration of the mitotic arrest (Bekier et al., 2009; Huang et al., 2009), whereas exit from mitosis generally reduces sensitivity to anti-mitotic drugs (Brito and Rieder, 2006; Gascoigne and Taylor, 2008). Apoptosis can still occur after release from a prolonged delay in mitosis after longer-term changes, including activation of signalling pathways and new protein expression (Colin et al., 2015; Uetake and Sluder, 2010).

The intrinsic apoptotic pathway is activated when cytochrome *c* is released from mitochondria into the cytosol, where it forms a complex with Apaf-1 leading to the recruitment and activation of caspase-9, a cystyl-aspartame endoprotease. Caspase-9 in turn cleaves and activates the effector caspases-3 and -7, which act on multiple substrates to bring about the cellular changes associated with apoptosis, including cellular blebbing, chromatin condensation and internucleosomal DNA fragmentation (Budihardjo et al., 1999). Apoptosis is controlled during mitosis by protein phosphorylation and the destruction of regulators mediated by the ubiquitin proteasome pathway; these mechanisms couple the control of apoptosis to the progression of mitosis (Clarke and Allan, 2009). Caspase-9 is phosphorylated at an inhibitory site in mitosis by CDK1–cyclin-B1, the major mitotic protein kinase, which thereby restrains apoptosis during normal mitosis and the initial stages of mitotic arrest. If metaphase is not successfully resolved, then apoptosis is initiated during a prolonged mitotic arrest when the apoptotic signal overcomes the threshold set by caspase-9 phosphorylation (Allan and Clarke, 2007). Conversely, the apoptotic signal is initiated when phosphorylation of the anti-apoptotic protein Mcl-1 at T92 by CDK1–cyclin-B1 causes it to be degraded during a delay in mitosis (Harley et al., 2010; Wertz et al., 2011). Stabilisation of Mcl-1 by abolition of T92 phosphorylation or mutation of a destruction box (D-box) that is recognised by the APC/C inhibits apoptosis induced by microtubule poisons (Harley et al., 2010). In addition, the related anti-apoptotic proteins Bcl-2 and Bcl-x_L_ (encoded by *BCL2L1*) are phosphorylated and appear to be partially inhibited during mitosis (Terrano et al., 2010). The slow degradation of cyclin B1 even when the spindle assembly checkpoint is active can lead eventually to inactivation of CDK1–cyclin-B1 and slippage out of mitosis (Brito and Rieder, 2006). Whether or not apoptosis is initiated during mitotic arrest is likely to depend upon relative changes in the activities of CDK1–cyclin-B1 and regulators of apoptosis during the mitotic arrest (Allan and Clarke, 2007; Gascoigne and Taylor, 2008).

Although caspase-3 and caspase-7 do not appear to be regulated directly during mitosis by phosphorylation, these enzymes might also be controlled through interacting proteins. One candidate is X-linked inhibitor of apoptosis (XIAP), a protein of the IAP family that is capable of inhibiting activated caspases-3, -7 and -9 by direct binding (Deveraux et al., 1999; Takahashi et al., 1998). XIAP can also function as an E3 ubiquitin ligase and as a signal transduction intermediate (Eckelman et al., 2006; Lu et al., 2007). XIAP is overexpressed in a number of cancer cell lines, and its overexpression correlates with increased resistance to chemotherapeutic drugs, including inhibitors of mitosis (Durie et al., 2011; Holcik et al., 2000; Tamm et al., 2000).

XIAP is regulated acutely by post-translational modifications that can determine sensitivity to cellular stresses: phosphorylation at S87 by Akt proteins reduces XIAP auto-ubiquitylation and stabilises the protein in cisplatin-treated cells (Dan et al., 2004), whereas phosphorylation at S430 by TANK-binding kinase 1 (TBK1) or the IκB kinase IKKε results in the auto-ubiquitylation and subsequent proteasomal degradation of XIAP after viral infection of cells (Nakhaei et al., 2012). However, it has been unclear whether regulation of XIAP plays a role in controlling apoptosis during mitosis. In this study, we show that XIAP is phosphorylated at specific sites during mitosis by CDK1–cyclin-B1, which inhibits the anti-apoptotic activity of XIAP and makes mitotic cells more sensitive to pro-apoptotic signals. Thus, XIAP plays a key role in coupling the control of apoptosis to the progression of mitosis.

## RESULTS

### XIAP restrains apoptosis in response to prolonged mitotic arrest

**Fig. 1.**
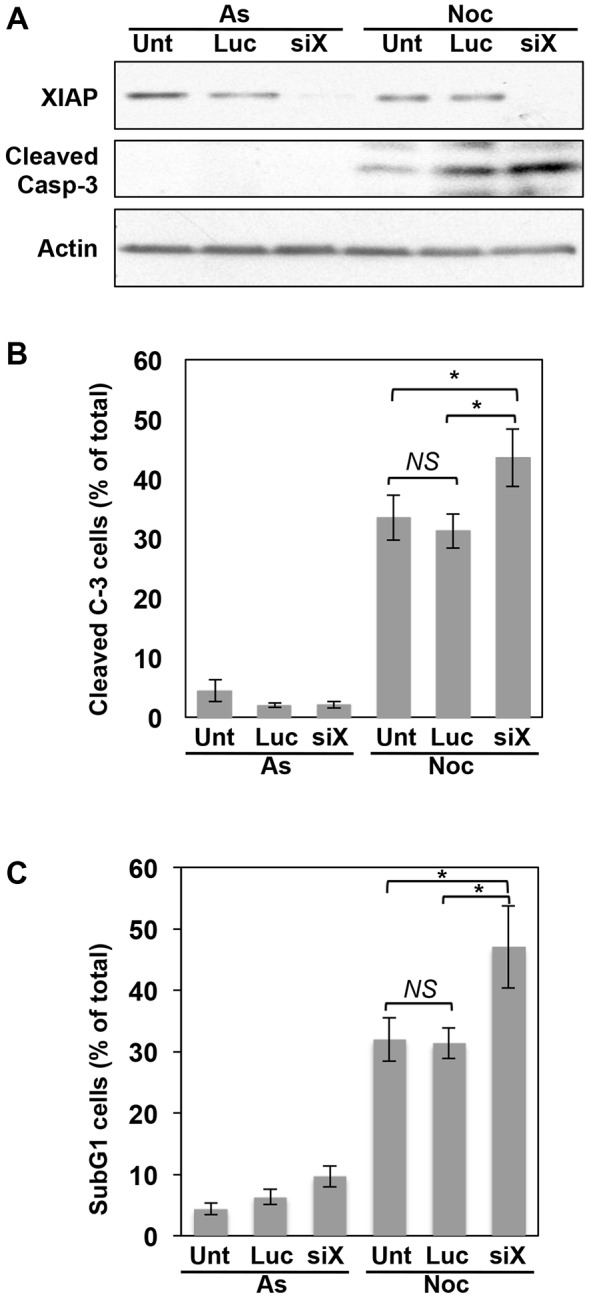
**Endogenous XIAP restrains apoptosis in response to mitotic arrest.** Knockdown of endogenous XIAP results in increased caspase-3 activity and apoptosis in response to prolonged mitotic arrest. U2OS cells were either untransfected (Unt), transfected with siRNA directed against luciferase, as control (Luc), or against XIAP (siX) for 48 h and then either untreated (asynchronous, As) or treated with 250 ng/ml nocodazole for a further 48 h (Noc). (A,B) Generation of active, cleaved caspase-3 was analysed by (A) SDS-PAGE and western blotting, or (B) fluorescence microscopy. In B, at least 300 cells were counted for each category in every experiment (*n*=4). (C) Apoptosis was analysed by sub-G1 DNA content by flow cytometry (*n*=3). Results in B and C represent mean±s.d. *P<0.05; *NS*, non-significant difference (Student's unpaired *t*-test).

We tested the role of human XIAP in the control of apoptosis in response to the disruption of mitosis. We found that knockdown of XIAP in U2OS cells by small interfering RNA (siRNA) promoted the cleavage and activation of caspase-3, a major effector of apoptosis (Fig. 1A,B; Fig. S1A), and caused sub-G1 apoptotic fragmentation of DNA (Fig. 1C; Fig. S1B) after prolonged treatment of the cells with nocodazole, a microtubule poison that prevents mitotic spindle assembly and arrests cells in mitosis. Entirely consistent with these results, there was increased active caspase-3 and sub-G1 DNA fragmentation in XIAP^−/−^ HCT116 cells compared to that seen in wild-type cells after prolonged treatment with nocodazole (Fig. S1C,D).

### XIAP is phosphorylated during mitosis

**Fig. 2.**
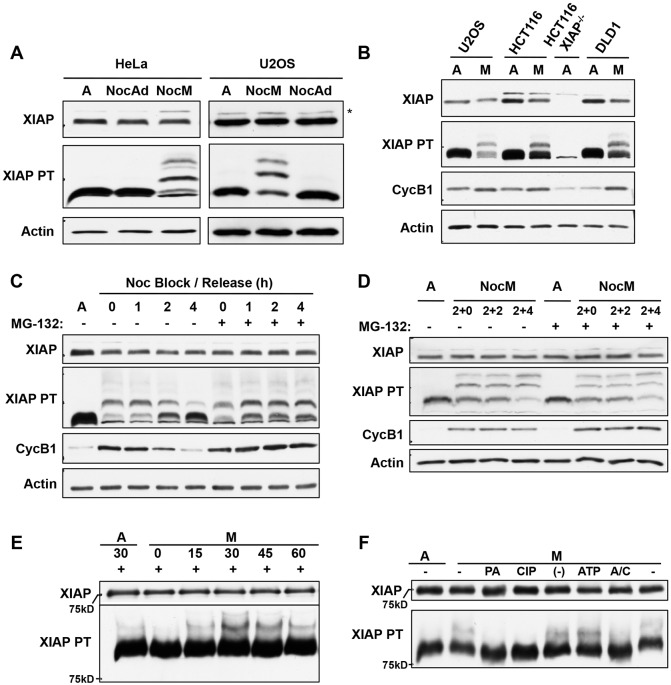
**XIAP is phosphorylated in mitotically arrested cells.** (A) Analysis of XIAP phosphorylation on PhosTag gels. HeLa and U2OS cells were treated with 100 ng/ml nocodazole for 17 h and harvested as either non-adherent nocodazole mitotic (NocM) or nocodazole adherent interphase (NocAd) samples. Untreated asynchronous cells (labelled A) were used as a control. Samples were analysed by SDS-PAGE with or without the addition of PhosTag (PT) as indicated followed by western blotting using the indicated antibodies. *denotes a non-specific band detected by the anti-XIAP antibody. (B) XIAP is phosphorylated in mitotically arrested cells. U2OS, HCT116 and DLD-1 cells were treated with 100 ng/ml nocodazole for 17 h. The non-adherent nocodazole-treated mitotic (M) samples were collected and analysed in comparison with samples from untreated asynchronous cells (labelled A), and analysed. (C) XIAP phosphorylation is mitosis specific and the protein is dephosphorylated as cells exit mitosis. U2OS cells were either untreated (labelled A) or treated with 100 ng/ml nocodazole for 17 h before the rounded up cells were either lysed, or re-plated in nocodazole-free medium in the absence or presence of 10 μM of the proteasome inhibitor MG132 for a further 1, 2 or 4 h. (D) XIAP is continuously phosphorylated during mitosis and the phosphorylated form is stable. U2OS cells were either untreated (labelled A) or pre-treated with 10 μM MG132 for 2 h, before adding 100 ng/ml nocodazole to the medium and leaving for 2 h. The rounded up mitotic cells were then isolated, and either lysed or re-plated in the same medium for a further 2 or 4 h. The floating cells were collected at the end of the timecourse. (E) Recombinant GST–XIAP is phosphorylated in mitotic cell extract. Asynchronous (labelled A) and nocodazole-treated mitotic (M) HeLa cell extracts were incubated with recombinant GST-tagged XIAP protein for the indicated time. (F) Phosphorylation of GST–XIAP is inhibited by a CDK inhibitor or phosphatase. *In vitro* phosphorylation reaction in mitotic (M) cell extracts was carried out for 30 min in the presence of 10 μM purvalanol A (PA), 0.4 U calf intestinal phosphatase (CIP), phosphatase buffer (–), an ATP-regenerating system (ATP) or both an ATP-regenerating system and CIP (A/C). A lysate prepared from untreated asynchronous cells (labelled A) was used as a control.

These results established that XIAP plays a role in the control of apoptosis in response to prolonged mitotic arrest. We did not, however, observe any periodic changes in the levels of the protein during the cell cycle (Fig. S2A). Similarly, the level of XIAP was maintained during a prolonged mitotic arrest until exit from mitosis and the induction of apoptosis (Fig. S2B). To determine whether XIAP is regulated post-translationally by phosphorylation during mitosis, we treated HeLa and U2OS cells with nocodazole and compared isolated rounded-up mitotic cells with adherent interphase cells. Modification of XIAP leading to retardation of the protein was not readily detected on western blots of conventional SDS-PAGE gels (Fig. 2A). However, modification of XIAP in mitotic cells was clearly apparent when gels were supplemented with PhosTag, an acrylamide-bound ligand that preferentially retards the migration of phosphorylated proteins (Kinoshita et al., 2006). There was one major and one minor retarded form of XIAP in rounded-up mitotic cells that was not present in adherent interphase cells also treated with nocodazole nor in untreated asynchronous cells, demonstrating that XIAP was phosphorylated on at least two sites specifically during mitosis, with one site predominating (Fig. 2A). XIAP was also phosphorylated in mitotic HCT116 and DLD1 human colorectal cancer cells with a very similar pattern of phosphorylated forms (Fig. 2B).

The mitotic phosphorylation of XIAP was reversed in parallel with cyclin B1 degradation when U2OS cells were released from mitotic arrest by washing out nocodazole. Dephosphorylation of XIAP was prevented by the proteasome inhibitor MG132, which prevents the degradation of cyclin B1 even in the absence of the checkpoint signal and maintains cells in mitosis (Fig. 2C). When mitotically arrested cells were maintained in nocodazole having been synchronised in the period of the arrest, phosphorylated forms of XIAP progressively accumulated over 2–6 h. MG132 did not alter the pattern of phosphorylated forms during mitotic arrest, indicating that both hypo- and hyper-phosphorylated XIAP were stable during the period of arrest (Fig. 2D).

Purified recombinant XIAP expressed as a fusion protein with glutathione-S-transferase (GST–XIAP) was also phosphorylated in a mitotic HeLa cell extract, with one major retarded form observed on PhosTag gels that accumulated over 30 min (Fig. 2E). Formation of phosphorylated XIAP form was inhibited by calf intestinal phosphatase (CIP) or upon inhibition of cyclin-dependent kinases (CDKs) by purvalanol A (Fig. 2F), indicating that mitotic phosphorylation of this major site is dependent on CDK1 in complex with cyclin B1 rather than cyclin A, which is lost from mitotically arrested cells prior to preparation of the extract.

### Identification of sites of mitotic phosphorylation in XIAP

**Fig. 3.**
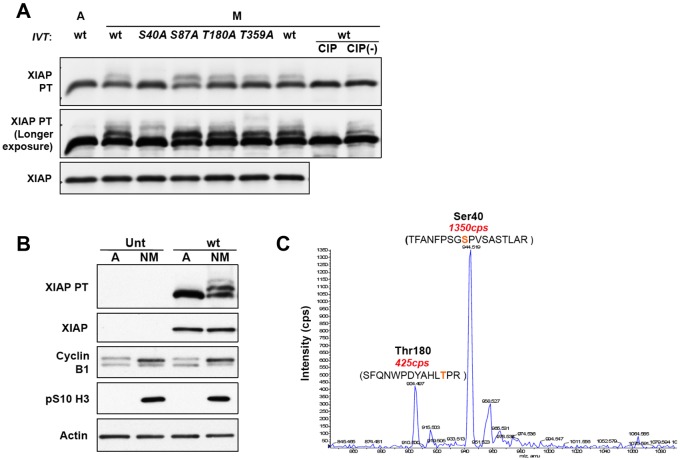
**Identification of sites in XIAP phosphorylated in mitosis.** (A) *In vitro* translated (IVT) XIAP is phosphorylated in mitotic cell extract. Wild-type (wt) and four mutant XIAP proteins were expressed *in vitro* to a similar level by IVT and added to extracts from asynchronous (labelled A) or mitotically arrested HeLa cells to a level much greater than endogenous XIAP protein. Nocodazole-treated mitotic extract (M) was also incubated with wild-type protein in the presence of calf intestinal phosphatase (CIP) or phosphatase storage buffer [CIP(−)] to confirm the phosphorylation. Reactions were terminated by the addition of 2× SDS lysis buffer, and the samples were analysed by SDS-PAGE with or without the addition of PhosTag (PT) as indicated followed by western blotting. (B,C) GFP-tagged XIAP is mitotically phosphorylated at S40. (B) U2OS cells that were either untransfected (Unt) or stably expressing GFP-tagged wild-type XIAP (wt) were treated with 100 ng/ml nocodazole for 17 h. The floating mitotic cells were collected at the end of the treatment and analysed by PhosTag SDS-PAGE and western blotting. (C) GFP–XIAP was precipitated from 2.2 mg cell lysate of mitotically arrested U2OS XIAP wild-type cells that had been treated with 100 ng/ml nocodazole for 24 h. GFP–XIAP was immunoprecipitated using GFP-Trap beads and analysed by mass spectrometry of tryptic peptides.

Human XIAP contains four serine and threonine residues (S40, S87, T180 and T359) that are followed immediately by a proline residue, a characteristic of phosphorylation sites targeted by proline-directed kinases such as CDK1–cyclin-B1. S40 has been identified in a global analysis of phosphorylation sites (Mertins et al., 2013) and S87 has been shown to be phosphorylated by Akt proteins (Dan et al., 2004). To analyse these potential mitotic phosphorylation sites, we mutated each residue to a non-phosphorylatable alanine residue and produced the resulting proteins by *in vitro* transcription and translation (IVT) in mammalian reticulocyte lysate. When incubated in mitotic HeLa cell extract, the wild-type, S87A, T180A and T359A proteins were all phosphorylated whereas mutation of S40 abolished the formation of the predominant phosphorylated form (Fig. 3A), indicating that this residue was the major phosphorylation site.

In another approach to determine the site(s) of mitotic phosphorylation, XIAP stably expressed as a fusion with green fluorescent protein (GFP–XIAP) was immunoprecipitated from mitotic nocodazole-treated U2OS cells. We confirmed that the protein was indeed phosphorylated in mitosis, exhibiting one major and one minor phosphorylated form (Fig. 3B), before analysis of tryptic peptides by nano liquid chromatography tandem mass spectrometry (nano LC-MS/MS) on a LTQ-Obitrap Velos mass spectrometer in combination with a neutral loss scan on a 4000 QTRAP mass spectrometer to detect phosphorylated peptides. Two phosphorylation sites were identified, with a major peptide phosphorylated on S40 and a less abundant peptide phosphorylated on T180 (Fig. 3C). Taking into account the proportion of modified XIAP detected on PhosTag gels, this data shows that ∼50% of XIAP is phosphorylated at S40 in cells arrested in mitosis, with additional phosphorylation at T180 at a lower stoichiometry.

### XIAP is phosphorylated at S40 in mitosis

**Fig. 4.**
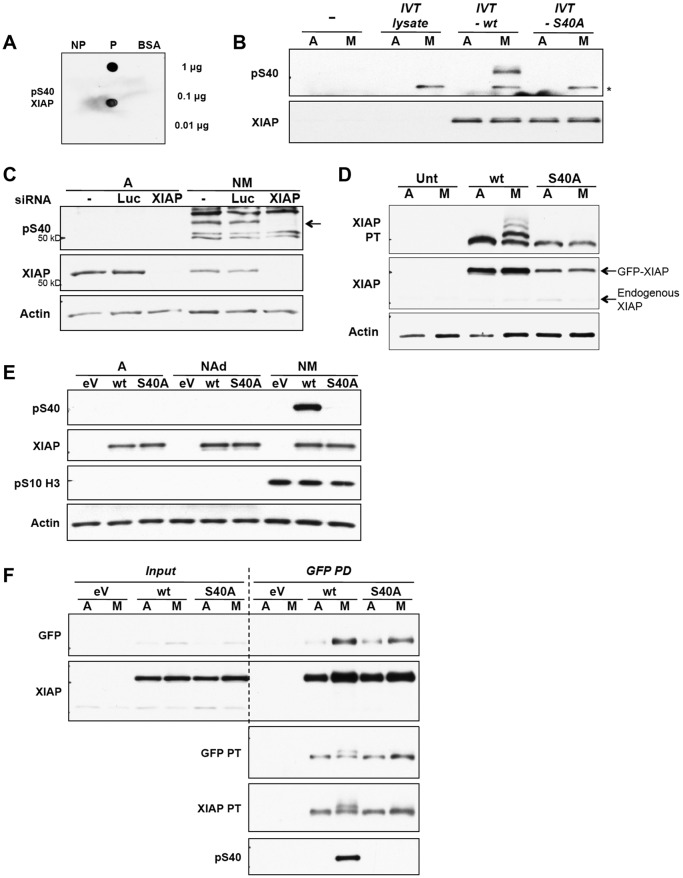
**XIAP is phosphorylated at S40 in mitotic cells.** (A) An antibody generated against pS40 XIAP is specific for the phosphopeptide. Decreasing amounts of the non-phospho-peptide (NP), phospho-peptide (P) and BSA control were spotted onto nitrocellulose membranes. The membranes were immunoblotted with 1:500 of the diluted phospho-antibody purified from serum. (B) The pS40 antibody specifically recognises XIAP phosphorylated at S40. Wild-type (wt) and S40A mutant XIAP protein were generated using IVT, and the reaction mix for each protein was incubated with either asynchronous (labelled A) or mitotic arrested (M) HeLa cell extract. Samples were analysed by SDS-PAGE and western blotting. The IVT reaction mix incubated without vector DNA was used as an extra negative control (IVT lysate), which showed that the non-specific band (*) was a protein present in the reticulocyte lysate that was phosphorylated by HeLa mitotic extract. (C) Endogenous XIAP is phosphorylated at S40 in cells. U2OS cells were transfected with siRNA against XIAP or Luciferase (Luc). At 48 h post transfection, cells were either untreated (labelled A) or treated with 250 ng/ml nocodazole for 24 h (NM). All cells were collected from untreated samples and the floating cells were collected from nocodazole-treated samples. Cells were lysed, analysed by SDS-PAGE and immunoblotted with the anti-pS40 antibody. Phosphorylated XIAP, which is ablated by the specific siRNA, is indicated by an arrow. (D–F) S40 is required for XIAP phosphorylation in cells. U2OS cells which were untransfected (Unt) or stably expressing GFP–XIAP wild-type (wt) or S40A were either untreated (labelled A) or treated with 100 ng/ml nocodazole (M) for 17 h. The floating cells were collected from nocodazole-treated samples. All cell lysates were analysed by SDS-PAGE and immunoblotted with indicated antibodies. In D, samples were analysed on a PhosTag (PT) gel. In E, adherent interphase cells (NAd) from nocodazole-treated samples were also analysed. In F, cell lysates were incubated with GFP-Trap beads and GFP-tagged proteins were immunoprecipitated (pulldown, PD) from the samples. Lysates that were not incubated with beads were used as input samples. eV, empty vector.

To facilitate analysis of the phosphorylation of S40 in XIAP, a specific polyclonal antibody was raised by inoculation with a synthetic peptide that included the phosphorylated site. Antibodies were purified from serum by negative selection against the dephosphorylated peptide followed by positive selection against the phosphopeptide (denoted the pS40 antibody). Selectivity was tested on dot blots where bovine serum albumin (BSA)-conjugated peptides were probed using purified antibodies (Fig. 4A). The pS40 antibody detected IVT XIAP that had been phosphorylated in mitotic HeLa cell extracts, whereas the S40A mutant was not detected. Thus, the antibody is specific for phosphorylated S40 and no other phosphorylation site in XIAP (Fig. 4B). Endogenous XIAP phosphorylated on S40 was detected in U2OS cells arrested in mitosis with nocodazole; the specific polypeptide was identified through its disappearance upon prior depletion of XIAP by siRNA transfection (Fig. 4C).

We confirmed that S40 was phosphorylated in GFP–XIAP expressed in U2OS cells arrested in mitosis with nocodazole. Analysis of the migration of GFP–XIAP on PhosTag gels (Fig. 4D) showed that mutation of S40 inhibited the formation of not only the major phosphorylated form but also other minor phosphorylated forms. This indicates that S40 is necessary for phosphorylation at one or more of the less abundant sites, most likely including T180. GFP–XIAP was phosphorylated on S40 in mitotic U2OS cells but not in untreated adherent interphase cells or nocodazole-treated adherent cells (Fig. 4E). We unequivocally identified this phosphorylated form as GFP–XIAP by its immunoprecipitation from cell extracts prior to western blotting (Fig. 4F).

### XIAP is phosphorylated at S40 by CDK1–cyclin-B1

**Fig. 5.**
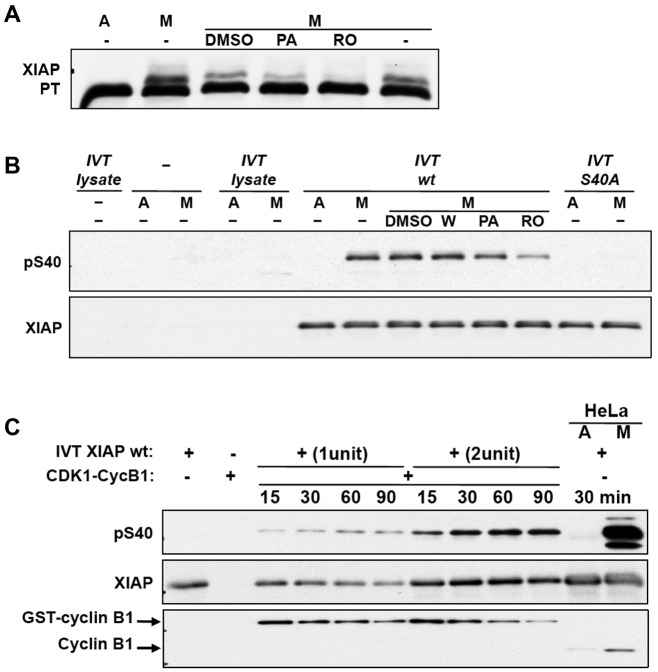
**XIAP is phosphorylated at S40 by CDK1–cyclin-B1.** (A) CDK1 kinase inhibitors suppress phosphorylation of XIAP *in vitro*. Wild-type XIAP produced by coupled *in vitro* translation and transcription (IVT XIAP) was incubated with asynchronous (labelled A) or mitotic nocodazole-treated (M) HeLa cell extracts with or without kinase inhibitors purvalanol A (PA, 10 μM) and RO 3306 (RO, 10 μM). Lysates were analysed by PhosTag (PT) SDS-PAGE and immunoblotted with anti-XIAP antibody. (B) Wild-type (wt) IVT XIAP was incubated with asynchronous (labelled A) or mitotic nocodazole-treated (M) HeLa cell extracts at 30°C for 30 min with or without kinase inhibitors. Wortmannin (W) was used at 0.1 μM, and PA and RO were used as in A. IVT reticulocyte lysate without vector DNA, HeLa extracts incubated alone, IVT lysate without vector DNA incubated with HeLa extracts and IVT S40A mutant incubated with HeLa extracts were used as negative controls. Reactions were analysed by SDS-PAGE and immunoblotted with pS40 XIAP antibody. (C) S40 of XIAP is phosphorylated by CDK1–cyclin-B1. Active recombinant GST-tagged CDK1–cyclin-B1 was incubated with two different amounts of wild-type IVT XIAP at 30°C for various times as indicated. IVT XIAP alone, CDK1–cyclin-B1 alone and IVT XIAP incubated with HeLa extracts were used as controls. Reactions were stopped by the addition of 2× SDS lysis buffer. Samples were analysed by SDS-PAGE and western blotting.

The previous observation that purvalanol A inhibited the phosphorylation of GST–XIAP in mitotic HeLa cell extract (Fig. 2F) indicated that phosphorylation was dependent on a CDK1–cyclin-B1. Similarly, phosphorylation of IVT XIAP incubated in mitotic HeLa cell extract and analysed on a PhosTag gel (Fig. 5A) was inhibited by purvalanol A and RO3306, a more selective inhibitor that specifically targets CDK1 and not DYRK1A at the concentration used (Vassilev et al., 2006). We confirmed that CDK inhibitors, but not other kinase inhibitors, including wortmannin, inhibited the phosphorylation of S40 in XIAP in mitotic extracts (Fig. 5B). Futhermore, XIAP was phosphorylated on S40 in a time-dependent manner upon incubation with purified CDK1–cyclin-B1 (Fig. 5C), showing that this kinase catalyses the phosphorylation of XIAP directly.

### Mutation of S40 to an aspartate residue inhibits XIAP binding to active effector caspases

**Fig. 6.**
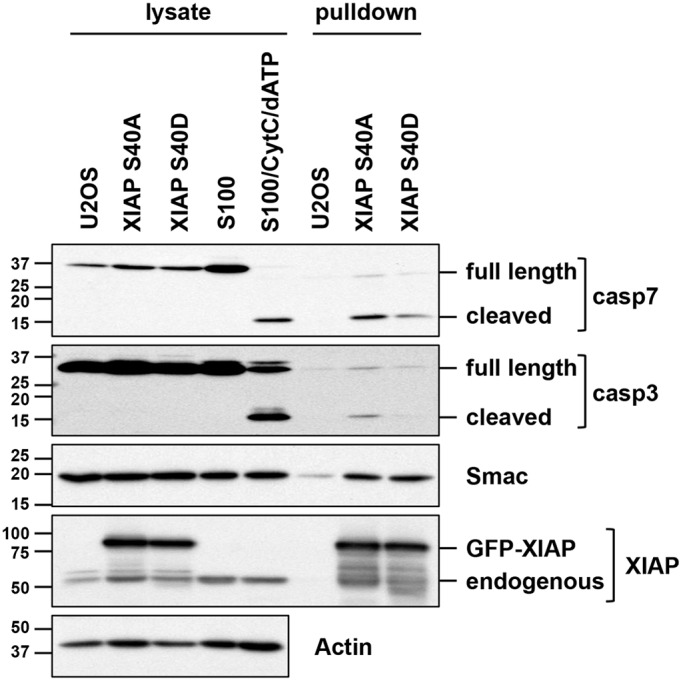
**The ability of XIAP to bind cleaved effector caspases is inhibited by mutation of S40 to a phosphomimetic aspartate residue.** HeLa S100 extract was incubated with cytochrome *c* and dATP to induce caspase cleavage prior to incubation with GFP–XIAP precipitated from cells stably expressing GFP–XIAP S40A or S40D protein. GFP–XIAP was recovered from the extract and co-precipitating proteins were detected by western blotting using the indicated antibodies. Cell lysates and HeLa cell S100 reactions were analysed for comparison.

XIAP is perhaps best known for its ability to bind to and inhibit activate caspases (Deveraux et al., 1999; Takahashi et al., 1998). To test whether mitotic phosphorylation of XIAP at S40 could affect this function, we carried out an *in vitro* binding assay utilising a potentially phosphomimetic aspartate mutant (S40D) as a surrogate for phosphorylated XIAP and comparing its ability to bind cleaved effector caspases with that of the non-phosphorylatable mutant S40A. GFP–XIAP S40D and S40A proteins produced in stably transfected U2OS cells were incubated with HeLa cell extract (S100) pretreated with cytochrome *c* and dATP to generate cleaved caspases (Li et al., 1997). Western blotting analysis of proteins co-precipitating from the extract demonstrated that XIAP S40D was less efficient at binding to cleaved caspase-7 and cleaved caspase-3 than the S40A protein (Fig. 6). In contrast, both XIAP proteins exhibited a similar ability to bind to Smac (also known as DIABLO), a protein that binds to and neutralises XIAP function (Du et al., 2000; Verhagen et al., 2000). This shows that the S40D protein is competent for some aspects of XIAP function but is inhibited in its ability to bind activated effector caspases, indicating that mitotic phosphorylation of XIAP at S40 restrains its ability to inhibit caspase activity.

### Modification of XIAP at S40 inhibits its anti-apoptotic activity

**Fig. 7.**
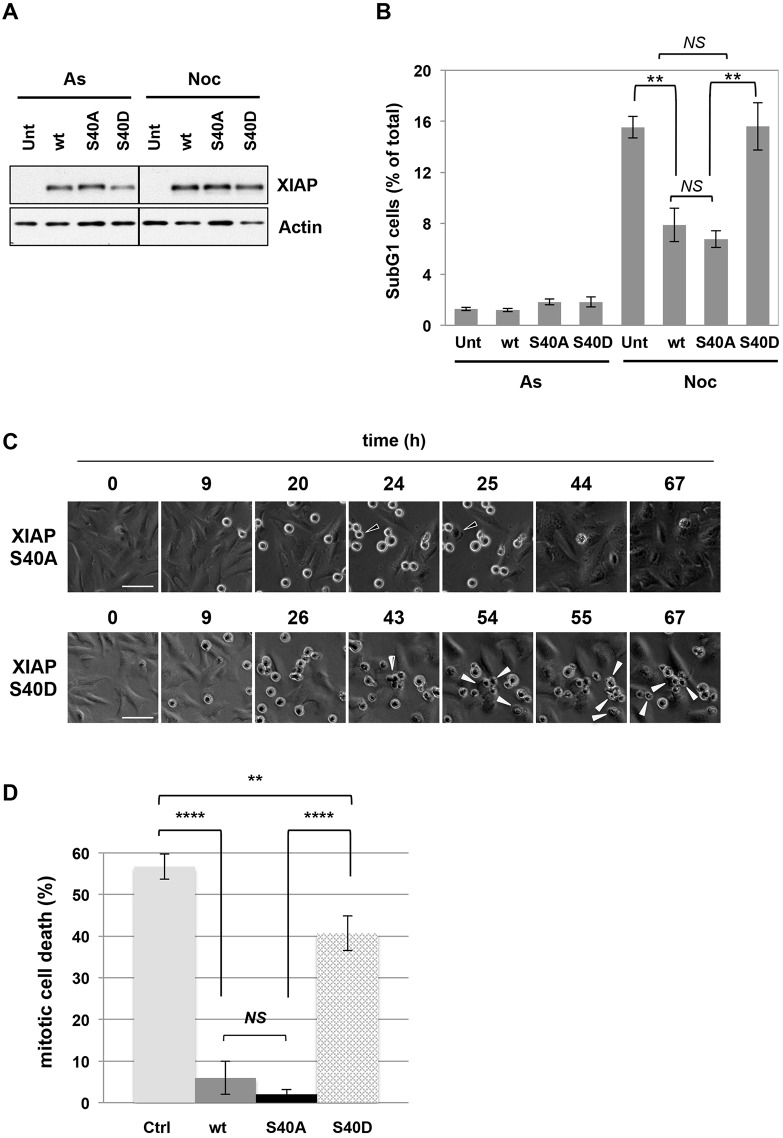
**Mutation of S40 in XIAP to a phosphomimetic aspartate residue inhibits its ability to protect cells against apoptosis induced by prolonged mitotic arrest.** (A,B) Cell death following mitotic arrest is not inhibited by expression of XIAP S40D protein. U2OS cells that were untransfected (Unt), stably expressing GFP-tagged wild-type (wt), S40A or S40D mutant XIAP protein were treated with 250 ng/ml nocodazole for 48 h. All cells were collected at the end of the timecourse and either (A) lysed, analysed by SDS-PAGE and western blotting (representative blots are shown) or (B) fixed and stained with propidium iodide for sub-G1 analysis using flow cytometry (*n*=3). In B, results are mean±s.d. **P<0.005; *NS*, non-significant difference (Student's unpaired *t*-test). (C,D) XIAP S40D fails to restrain apoptosis during mitotic arrest. Cells stably expressing GFP-tagged wild-type (wt), S40A or S40D mutant XIAP protein were treated with 250 ng/ml nocodazole. Cell fate following mitotic entry was monitored by time-lapse microscopy. (C) Representative images of cells expressing S40A or S40D mutant XIAP are shown. Examples of cells undergoing slippage (open arrows) or mitotic cell death (full arrows) are indicated. Scale bars: 50 µm. (D) Quantification of mitotic cell death in cell lines depicted in C, control cells (Ctrl) and cells expressing wild-type XIAP (wt). For each experiment (*n*=3), the fate of 50 cells was assessed. Results are mean±s.d. **P<0.01; ****P<0.0001; *NS*, non-significant difference (Student's unpaired *t*-test).

The inhibitory effect of the S40D mutation on the ability of XIAP to bind active caspases strongly suggests that phosphorylation of S40 in XIAP regulates its anti-apoptotic activity. To test this possibility, we first characterised the stability of wild-type and phosphorylation site mutant GFP–XIAP proteins during interphase and mitotic arrest. We found that GFP–XIAP S40A and S40D proteins, like the wild-type protein, were expressed at a constant level during mitotic arrest, indicating that the stability of the protein during mitosis was not affected by phosphorylation at this site (Fig. 7A). Consistent with results discussed above (Fig. 2D), phosphorylation of S40 detected by the specific antibody gradually increased over 2–6 h of arrest (Fig. S3). Importantly, whereas expression of either the wild-type XIAP or the S40A mutant strongly inhibited the induction of apoptosis after prolonged treatment with nocodazole, the S40D mutant was much less effective and produced no apparent inhibition of apoptosis (Fig. 7B; Fig. S4A). The difference between the S40A and S40D proteins strongly suggests that phosphorylation of S40 inhibits the anti-apoptotic activity of XIAP. The similar effect of wild-type and S40A XIAP expression is consistent with the dominant inhibitory activity of the non-phosphorylated protein, which is strongly elevated in both cases.

To determine whether modification of XIAP at S40 affects the acute apoptotic response during mitosis when phosphorylation occurs, we analysed the induction of cell death in individual mitotic cells by live-cell imaging. U2OS cells arrested in mitosis by 250 ng/ml nocodazole were tracked for around 70 h after entry into mitosis, by which time they had either slipped from mitosis, blebbing transiently and flattening down as they entered interphase (denoted survival), or had undergone rigorous blebbing followed by stasis (mitotic cell death) (Fig. 7C; Fig. S4B). About 55% of control cells transfected with an empty vector underwent mitotic cell death and cells that expressed the S40D mutant of XIAP showed only a slight reduction in cell death to 40% of control. By contrast, cells expressing either wild-type of S40A mutant of XIAP showed a strong reduction in mitotic cell death (<5% of control) (Fig. 7D), indicating that phosphorylation of XIAP at S40 inhibits its activity during mitosis and promotes mitotic cell death.

## DISCUSSION

The induction of apoptosis during mitosis provides a mechanism to selectively kill rogue cells that have failed to undergo chromosome segregation successfully and on schedule. This pathway also provides opportunities for the selective killing of cancer cells through drugs that interfere with mitosis, including microtubule poisons such as taxanes that prevent the proper attachment of chromosomes to spindle microtubules and cause a prolonged arrest in mitosis due to the activity of the spindle assembly (mitotic) checkpoint. Importantly, there are distinct post-translational mechanisms that control apoptosis during mitosis that facilitate drug-induced cell killing, perhaps especially in cancer cells that have sustained cellular stress but in which apoptosis is actively suppressed.

**Fig. 8.**
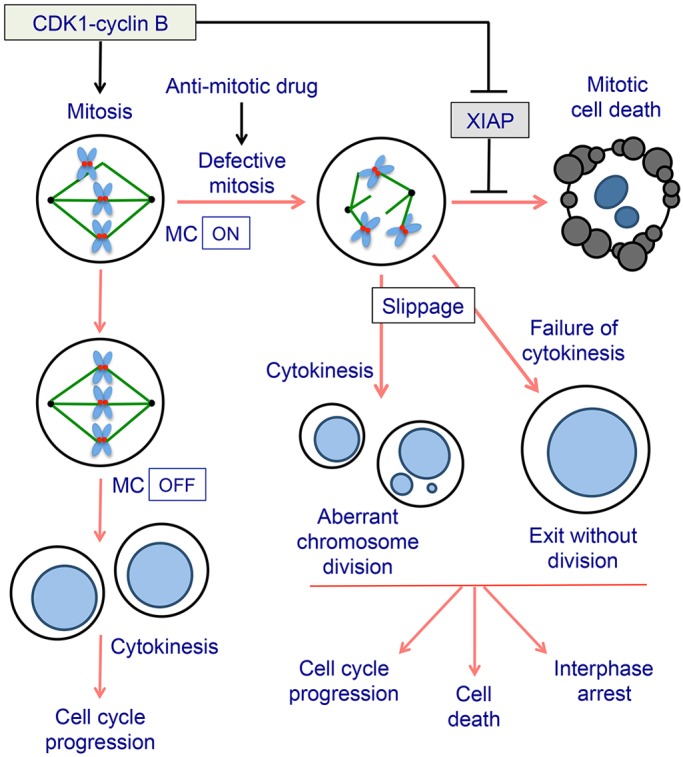
**Role of XIAP in determining cell fate during mitotic arrest.** CDK1–cyclin-B1 drives cells into mitosis and controls the induction of apoptosis by inhibitory phosphorylation of XIAP. After the last unattached kinetochore is correctly attached to spindle microtubules and the chromosomes are properly aligned, the spindle assembly or mitotic checkpoint (MC) is switched off. CDK1 is then inactivated as cyclin B1 is rapidly degraded, and cells progress through anaphase, undergo cytokinesis and exit mitosis. In the presence of an anti-mitotic drug, such as a microtubule poison, the checkpoint cannot be satisfied and cells are arrested for a prolonged period. Arrested cells might undergo cell death or can slip out of mitosis due to the slow degradation of cyclin B1 and undergo a variety of alternative cell fates. Phosphorylation of XIAP reduces the threshold for apoptosis during mitotic arrest. However, when a cell exits mitosis normally or slips out of mitotic arrest, XIAP is reactivated by dephosphorylation and contributes to cell survival.

In this report, we have identified a mechanism that controls the induction of cell death in mitotically arrested cells through the key regulator XIAP. Our results indicate that phosphorylation of XIAP at a single major site by the master mitotic kinase CDK1–cyclin-B negates the ability of XIAP to bind to active effector caspases and thereby reduces the threshold for apoptosis (Fig. 8). We show that ∼50% of the endogenous XIAP protein is phosphorylated at the inhibitory site S40 (Fig. 3), suggesting that the remaining protein that is not phosphorylated at this site continues to provide a restraint, albeit reduced, so that when XIAP is completely removed, apoptosis is increased further (Fig. 1). We propose that this mechanism is important for the control of cell death during mitotic arrest, because it reduces a downstream barrier to apoptosis once caspases-3 and -7 have been activated after proteolytic destruction of the upstream regulator Mcl-1 (Harley et al., 2010; Wertz et al., 2011). The time-dependent loss of Mcl-1 releases cytochrome *c* from mitochondria, resulting in a pro-apoptotic signal that overwhelms the brake set by inhibitory phosphorylation of caspase-9 (Allan and Clarke, 2007), which is also activated by dephosphorylation as mitotic arrest progresses (Harley et al., 2010). The inhibition of XIAP function by phosphorylation would make mitotically arrested cells less resistant to caspase activation and might explain the increased sensitivity of such cells to BH3 mimetics that inhibit Mcl-1, Bcl-2 and Bcl-x_L_ (Colin et al., 2015; Shi et al., 2011). If cells survive mitotic arrest and are released into interphase, dephosphorylation of XIAP would restore its anti-apoptotic function and raise the threshold for apoptosis again.

The sensitisation of mitotically arrested cells to apoptosis can be hypothesised to actively remove cells that become severely delayed in mitosis due to problems with chromosome segregation, supernumerary centrosomes or even aneuploidy. This would provide a mechanism to suppress the propagation of chromosome abnormalities (Fig. 8). Cancer cells exhibiting such defects, however, have presumably gone through a selection crisis in which the mechanism of apoptosis during mitosis is actively supressed. Two levels of the pathway at which this might well occur are upon the overexpression of the anti-apoptotic Bcl-2 family proteins, preventing cytochrome *c* release and caspase activation, or upon XIAP overexpression, blocking the action of caspases. At present it is unclear whether aberrant changes in the post-translational regulation of these components also contribute to the suppression of mitotic cell death in cancer cells, but if this is the case then targeting these mechanisms could activate apoptosis particularly well in such stressed cells.

The regulation of XIAP by CDK1–cyclin-B1 illustrates the close integration of the control of apoptosis with the progression of mitosis. In addition to XIAP, Mcl-1 is destroyed by proteolysis following phosphorylation by CDK1–cyclin-B1 (Harley et al., 2010; Wertz et al., 2011), and the related anti-apoptotic proteins Bcl-2 and Bcl-x_L_ might be directly inhibited by mitotic phosphorylation (Terrano et al., 2010). Opposing these pro-apoptotic events, we have shown that caspase-9 is inhibited by phosphorylation during normal mitosis and in the early stages of mitotic arrest (Allan and Clarke, 2007). The importance of the inhibition of XIAP by mitotic phosphorylation might be that it removes a downstream brake on apoptosis, resulting in one major point of regulation (caspase-9 activation) that permits the induction of apoptosis after a prolonged arrest. Anti-cancer drugs that target mitotic cells appear to exploit this mechanism to initiate mitosis by arresting or delaying them in mitosis long enough to overcome the temporal restraint to caspase activation. Cells that do not undergo full apoptosis from the mitotic state nevertheless carry a signal caused by caspase-dependent DNA damage that can subsequently determine long-term cell fate after release into interphase even though a higher threshold for apoptosis has been restored (Colin et al., 2015) (Fig. 8). Our results further emphasise that cell death during mitosis is a form of caspase-dependent apoptosis, albeit one under mitosis-specific post-translational controls.

## MATERIALS AND METHODS

### Cell culture and cell extracts

HeLa (Ohio) and U2OS (HTB96) cell lines were obtained from Cancer Research UK Laboratories, London, UK. HCT116 parental and XIAP-knockout cell lines were purchased from Horizon Discovery Ltd., Cambridge, UK. All cell lines were confirmed as negative for mycoplasma infection. Cells were cultured in Dulbecco's modified Eagle's medium (DMEM; Invitrogen) supplemented with 10% (v/v) fetal bovine serum (Biosera) and 1% (v/v) penicillin-streptomycin (Invitrogen) at 37°C and in 5% CO_2_. U2OS cells stably expressing GFP–XIAP proteins were generated by transfection with pEGFP-XIAP vector containing a G418 resistance marker. Cells were grown for 2–3 weeks with 800 µg/ml G418 Geneticin (Invitrogen) and maintained in 400 µg/ml G418. Cell extracts were prepared from mitotic or asynchronous HeLa cells at a protein concentration of 6–10 mg/ml in EBS (80 mM β-glycerophosphate-HCl pH 7.2, 20 mM EGTA, 15 mM MgCl_2_, 100 mM sucrose, 1 mM DTT, 1 mM PMSF) as described previously (Harley et al., 2010). HeLa cell cytosolic S100 extracts were supplied at a protein concentration of 8 mg/ml in extract buffer (10 mM Hepes-KOH pH 8.5, 10 mM KCl, 10 mM MgCl_2_, 0.5 mM DTT) (Computer Cell Culture Centre, Mons, Belgium).

### XIAP knock down by siRNA

Four RNA oligonucleotides that made up the siGENOME SMARTpool XIAP siRNA (Thermo Fisher Scientific) were tested individually. Two of the four oligonucleotides that resulted in the most efficient depletion of XIAP were used together in future experiments. The two XIAP siRNA oligonucleotides and the luciferase control siRNA were: XIAP siRNA 1, 5′-GCACGGAUCUUUACUUUG-3′; XIAP siRNA 2, 5′-GAACUGGGCAGGUUGUAGA-3′; and luciferase siRNA, 5′-CGUACGCGGAAUACUUCGATT-3′.

U2OS cells were transfected with the oligonucleotides using Lipofectamine 2000 (Invitrogen) and incubated for 48 h. 250 ng/ml nocodazole was then added for a further 48 h as required.

### Western blotting

Antibodies were used as described in Table S1.

### Generation of a polyclonal antibody specific for XIAP phosphorylated at S40

Rabbits were inoculated with the phosphorylated peptide FANFPSGS*PVSASTL (where S* represents the phosphorylated residue) by Moravian Biotechnology. A phospho-specific antibody, pS40 XIAP, was purified from the serum obtained after a second inoculation on a column of phosphorylated peptide coupled to Reactigel beads (Pierce Biotechnology), eluted with 0.1 M glycine-HCl (pH 2.5), which was neutralised with the addition of 1.5 M Tris-HCl (pH 8.5) supplemented with 100 µg/ml BSA, 50% glycerol and 0.05% sodium azide, and stored at −20°C.

Phosphorylated or non-phosphorylated peptide was coupled to immunoglobulin-free BSA (Sigma-Aldrich) using 50% (w/v) glutaraldehyde solution (Sigma-Aldrich) diluted in PBS. The BSA-conjugated peptide solutions were serially diluted in PBS, spotted onto nitrocellulose membrane and air-dried before being incubated in western blocking buffer. They were then incubated with 1:500 diluted phospho-specific antibody overnight at 4°C, followed by washes and secondary antibody probing.

### Detection of active caspase-3

Cells on coverslips or glass slides were permeabilised by incubating in ice-cold 80% ethanol for 15 min, and followed by two washes in PBS. Cells were blocked in 5% BSA and 0.2% Triton X-100 in PBS for 20 min at room temperature, before they were incubated with anti-cleaved caspase-3 antibody (cat. no. 9661, Cell Signaling) at a 1:400 dilution in 1% BSA and 0.2% Triton-X-100 in PBS at room temperature for 1 h. After two 5-min washes with 0.2% Triton X-100 in PBS on a rotary shaker, cells were incubated with 1:200 diluted TRITC-conjugated anti-rabbit-IgG secondary antibody (cat. no. R0156, Dako) and 250 ng/ml DAPI (Sigma) in 1% BSA and 0.2% Triton X-100 in PBS in the dark at room temperature for 1 h. This was followed by two 5-min washes with 0.2% Triton X-100 in PBS and one 5-min wash with PBS on a rotary shaker. Slides were mounted with mounting medium (Dako), air dried and kept at 4°C in the dark until they were viewed under the microscope.

### Microscopy

Fixed cells were visualised using Zeiss Axiovert 100 microscope with specific filters for the excitation of fluorophores. Images were taken at 63× magnification using Openlab software. For live-cell imaging, cells were imaged every 10 min in a heated chamber on a Zeiss Axiovert 200 M microscope. Images were acquired using a C4742-80-12AG camera (Hamamatsu) and µmanager software and were processed using ImageJ Fiji. Analysis was carried out on cells that were arrested in mitosis for at least 6 h with cell death being defined by cell morphology and cessation of movement.

### Flow cytometry

Cells stained with propidium iodide were analysed on a FACS flow cytometer (Becton Dickinson) on the FL3 channel. A minimum of 10^4^ cells were analysed for each sample. Data were displayed as dot plots and histograms showing the cell cycle phases and sub-G1 population using FlowJo software.

### GFP–XIAP precipitation from cells

U2OS cells stably expressing GFP or GFP–XIAP were treated with 100 ng/ml nocodazole for 17 h. The asynchronous cells from the untreated plate and the mitotically arrested cells from the nocodazole-treated plates were washed twice in cold PBS, and lysed in cold GFP lysis buffer (10 mM Tris-HCl pH 7.5, 150 mM NaCl, 0.5 mM EDTA, 5 mM β-glycerophosphate, 50 mM NaF, 1 mM Na_3_VO_4_, 1 mM PMSF, 1 µg/ml each of aprotinin, leupeptin and pepstatin A, 0.2% CHAPS). The protein concentration of the lysates was adjusted to 1 mg/ml with GFP lysis buffer. 300 µg lysate was mixed with 10 µl pre-washed GFP-Trap beads (ChromoTek) for each sample, and incubated at 4°C for 1 h on a rotator. The beads were pelleted by centrifugation at 4°C, washed once with GFP lysis buffer followed by two washes with 10 mM Tris-HCl (pH 7.5). Samples were analysed by SDS-PAGE and western blotting.

### *In vitro* phosphorylation assays

GST–XIAP was expressed from the pGEX-6P-1 vector in *E.coli* BLR(DE3) competent cells (Novagen), purified on Glutathione–Sepharose 4B beads (Amersham) and stored in 10 mM Hepes-KOH (pH 7.5), 150 mM NaCl, 0.07% β-mercaptoethanol. *In vitro* translated and transcribed (IVT) XIAP was produced using the T_N_T Quick Coupled Transcription/Translation system (Promega) using XIAP inserted in the pcDNA3.2/V5-DEST vector. Either 50 ng of GST–XIAP or 3 µl IVT XIAP was incubated with 1 µl mitotic HeLa EBS extract in phosphorylation reaction buffer (50 mM Tris-HCl pH 7.5, 1 mM DTT) plus MgATP (0.1 mM ATP, 10 mM MgCl_2_, 5 mM Hepes-KOH pH 7.5) at 30°C for 30 min in a total reaction volume of 10 µl. For phosphorylation by CDK1–cyclin-B1, 25 ng active recombinant kinase (Millipore) replaced HeLa extract. Where specified, 1 U/µl calf intestinal phosphatase (CIP) (Roche) and 1 µl of 10× CIP reaction buffer (0.5 M Tris-HCl pH 8.5, 1 mM EDTA), 1 µl of 10× ATP-regenerating system (0.1 mg/ml creatine kinase and 50 mM creatine phosphate) or kinase inhibitors were added. After incubation, reactions were stopped by addition of an equal volume of 2× SDS lysis buffer, 5% β-mercaptoethanol and 0.05% Bromophenol Blue. Owing to the presence of EGTA in the cell extract EBS buffer, which would interfere with PhosTag reagent, an equal molar concentration of MnCl_2_ was added to the loading samples that were going to be tested on PhosTag gels. Samples were boiled for 4 min, then analysed by SDS-PAGE and western blotting.

### *In vitro* binding assay

HeLa S100 extract (4 mg) was incubated with 10 µM cytochrome *c* and 1 mM dATP together with an ATP-regenerating system (1 mM ATP, 10 µg/ml creatine kinase and 5 mM creatine phosphate) at 30°C for 3 h. U2OS cells stably expressing GFP–XIAP S40A or S40D were lysed in GFP-Trap buffer (10 mM Tris-HCl pH 7.5, 150 mM NaCl, 0.5 mM EDTA and 0.5% NP40). A total of 2 mg lysate was diluted 2:3 with dilution buffer (GFP-Trap buffer without NP40) and incubated with 25 µl GFP-TrapM beads (ChromoTek), 4°C, 1 h with rotation. Beads were isolated using a magnet (BioRad) and washed three times in GFP-Trap buffer diluted as above followed by one wash in dilution buffer. GFP–XIAP beads were then incubated with S100, cytochrome *c* and dATP reactions at room temperature for 90 min with rotation. Beads were then isolated, washed as above and boiled in SDS gel-loading buffer.
